# Estimation of dental age based on the developmental stages of permanent teeth in Japanese children and adolescents

**DOI:** 10.1038/s41598-022-07304-2

**Published:** 2022-02-28

**Authors:** Katsuaki Kuremoto, Rena Okawa, Saaya Matayoshi, Kazuma Kokomoto, Kazuhiko Nakano

**Affiliations:** Department of Pediatric Dentistry, Osaka University Graduate School of Dentistry, 1-8 Yamada-oka, Suita, Osaka, 565-0871 Japan

**Keywords:** Forensic dentistry, Paediatric dentistry, Panoramic radiography

## Abstract

Assessment of children’s growth and development based on general and oral developmental status and dental age is important in pediatric dentistry for appropriate diagnosis and treatment. Teeth are a useful maturation index because they are unlikely to be affected by exogenous factors such as disease. We examined the correlation between chronological and dental age of permanent teeth in Japanese children and adolescents using orthopantomography. The sample comprised 1024 orthopantomographs from individuals aged 3–18 years, which were stored in an electronic media database for 10 years (2009–2019). We classified the developmental stages of each permanent tooth were classified into 11 stages, clarified the dental age for each developmental stage, and prepared a conversion table. Using the results, we compared the sequence and rate of development of each permanent tooth. We clarified the dental age of each permanent tooth from childhood to mid-adolescence and established a method for calculating the dental age of the whole jaw that is appropriate for modern Japanese individuals. We found that girls tended to form teeth at a faster rate than boys until puberty, but boys caught up with girls after puberty, suggesting that secondary sexual characteristics are involved in the rate of tooth formation.

## Introduction

Evaluation of growth and development in children based on general and oral developmental status and dental age is important for appropriate diagnosis and treatment decisions in pediatric dentistry^1^. To monitor physical development, several maturation indicators are assessed, such as hard tissues (e.g., bones and teeth), sexual maturity (e.g., testicular growth and menarche), and physical characteristics (e.g., height and weight)^2^. Oral development status is monitored by functional assessment of eating, speaking, and breathing, and by maturation indicators, such as morphological assessment of maxillofacial morphology and permanent tooth germ^3^. Although many physiological indices can be affected by exogenous environmental factors, teeth have attracted attention as a useful maturation indicator because they are less susceptible to the effects of factors such as systemic nutritional status and disease^4–6^.

Dental age can be calculated from the age of eruption or the developmental stage of permanent teeth^7,8^. The age of eruption of permanent teeth can be assessed directly by intraoral examination or indirectly by orthopantomography^9^. Evaluation based on the age of eruption of permanent teeth has a narrow range of application owing to the difficulty of clearly identifying the timing, and the fact that it cannot be applied to deciduous dentition^10^. Another problem is that the age of eruption of permanent teeth is easily affected by local factors such as crowding, ectopic eruption, ankylosis of deciduous teeth, and early extraction^11^. In contrast, the calculation of dental age from the developmental stage of permanent teeth is more widely applicable because it is less affected by local factors and can be used for a wide range of ages^12^.

There are several methods for assessing the developmental stages of permanent teeth. To estimate dental age, Schour and Massler^13^ categorized the stages of morphological change in teeth and dentition into infancy and early childhood, late childhood, and adolescence. However, this method is problematic because estimation is less accurate for ages 7–9 years and has a large error range for ages 6–14 years^14^. Haavikko’s method^8^ classifies the developmental stages of 32 permanent teeth on the left and right sides of the upper and lower jaws into 12 stages for evaluation; this enables the calculation of the age of all teeth using the dental age conversion table for each developmental stage. However, Haavikko’s study was conducted on Finns approximately 50 years ago, so it is unclear whether its findings can be compared with dental age data for modern Japanese individuals. In the method devised by Demirjian et al.^15^, the developmental stages of the seven permanent teeth on the left side of the lower jaw (excluding the third molars) are classified into eight stages and scored, and dental age calculated from the total score. This method overestimates dental age for ethnicities other than French Canadian^16^. Goya et al.^17^ conducted a study on the dental age of Japanese children using the Demirjian method. They concluded that the Demirjian standards are unsuitable for use with modern Japanese individuals, and that standards specific to the Japanese population need to be developed.

Because the standard values for dental age vary by region and human population, it is necessary to use assessment methods appropriate for each individual^12,18,19^. To the best of our knowledge, only one study has investigated dental age in Japanese children^17^. Since the method of Goya et al. was calculated using the Demirjian method and corrections, the direct dental age of each tooth type was not indicated. Therefore, this study was conducted to clarify the dental age for each developmental stage of each permanent tooth using orthopantomographs of modern Japanese children and adolescents, and to create a conversion table that can be used in daily clinical practice. Furthermore, using the results of this study, we compared the sequence and rate of development of each permanent tooth in modern Japanese children and adolescents.

## Results

### Investigation of the distribution of samples

#### Left–right difference in age distribution

The t-test of the difference in age distribution between the bilateral homonymous teeth of boys and girls showed no significant difference (that the lowest *p* > 0.05) *p*-value was for stage O (crypt, no calcification) maxillary third molars in girls, and that the *p*-values for all developmental stages ranged from 0.12 to 1.00. We concluded that there was no significant left–right difference for any developmental stages, so we combined the results for the left and right sides. These combined values were used for all subsequent analyses.

#### Age distribution in each developmental stage

A cumulative frequency distribution diagram of Crc (crown complete) and Rc (complete root formation) in anterior teeth is shown in Supplementary Fig. 1. The Crc of the lower central incisor of girls showed positive skewness, but the other curves were very close to normal. The distribution also showed that girls tended to be younger than boys. A cumulative frequency distribution diagram of Crc and Rc in premolars is shown in Supplementary Fig. 2. The Rc of the male upper first premolar showed negative skewness, but the other curves were very close to normal. There was no significant difference in the maximum frequency between boys and girls. A cumulative frequency distribution diagram of Crc and Rc in molars is shown in Supplementary Fig. 3. The Crc of the lower first molar showed positive skewness in both sexes, but the curves of the upper first molar and the upper and lower second molars were very close to normal. The Rc of the third molar showed negative skewness in both sexes, indicating that the Crc scatter was large for both sexes. The molar distributions showed that the dental age of girls with only the first molar was lower than that of boys.

### Average age of permanent teeth by developmental stage

#### Central incisor

The dental age by developmental stage of the anterior teeth was calculated (Table 1). The data range for the upper central incisor was Cr3/4 (3/4 crown formation) to Rc. There was no difference in dental age between boys and girls until R1/4 (1/4 root formation), at approximately 6 years of age. After R1/2 (1/2 root formation) at approximately 7 years of age, the dental age of girls was significantly lower than that of boys in this developmental stage. The data range for the lower central incisor was Cr3/4 to Rc (as for the upper); for Crc, R1/2, and R3/4 (3/4 root formation), the dental age of girls was significantly lower than that of boys.

**Table 1:** Average age of anterior teeth formation stages.

	Tooth stage	Upper			Lower		
		Male	Female	Sex difference	Male	Female	Sex difference
		Mean ± SD (years)	Mean ± SD (years)		Mean ± SD (years)	Mean ± SD (years)	
Central incisor	Cr3/4	4.0 ± 0.6	4.0 ± 0.7	*p* = 0.81	3.7 ± 0.5	3.6 ± 0.4	*p* = 0.21
	Crc	5.3 ± 0.8	5.0 ± 0.7	*p* = 0.059	4.6 ± 0.7	4.3 ± 0.7	*p* = 0.0024
	R1/4	6.4 ± 0.9	6.2 ± 0.8	*p* = 0.14	5.3 ± 0.7	5.2 ± 0.8	*p* = 0.26
	R1/2	7.4 ± 0.8	6.8 ± 0.8	*p* < 0.001	6.1 ± 0.8	5.7 ± 0.7	*p* = 0.011
	R3/4	8.0 ± 0.8	7.6 ± 0.7	*p* < 0.001	7.0 ± 0.8	6.5 ± 0.6	*p* < 0.001
	Rc	8.9 ± 0.9	8.4 ± 0.6	*p* < 0.001	7.9 ± 0.7	7.7 ± 0.7	*p* = 0.14
Lateral incisor	Cr1/2	3.7 ± 0.4	3.7 ± 0.6	*p* = 0.76	–	–	–
	Cr3/4	4.8 ± 0.7	4.3 ± 0.6	*p* < 0.001	4.2 ± 0.7	3.9 ± 0.6	*p* = 0.013
	Crc	6.0 ± 1.0	5.6 ± 0.7	*p* = 0.0025	5.1 ± 0.7	5.0 ± 0.8	*p* = 0.14
	R1/4	6.8 ± 0.7	6.7 ± 0.8	*p* = 0.18	6.0 ± 0.6	5.7 ± 0.7	*p* = 0.064
	R1/2	7.9 ± 0.8	7.4 ± 0.6	*p* < 0.001	6.9 ± 0.7	6.4 ± 0.5	*p* < 0.001
	R3/4	8.7 ± 0.7	8.1 ± 0.7	*p* < 0.001	7.7 ± 0.7	7.5 ± 0.6	*p* = 0.12
	Rc	9.7 ± 0.8	9.3 ± 0.9	*p* = 0.07	8.7 ± 0.9	8.3 ± 0.9	*p* = 0.0058
Canine	Cr1/2	4.0 ± 0.8	3.7 ± 0.5	*p* = 0.014	3.8 ± 0.5	3.7 ± 0.6	*p* = 0.27
	Cr3/4	5.2 ± 0.8	4.6 ± 0.8	*p* < 0.001	4.9 ± 0.9	4.4 ± 0.7	*p* < 0.001
	Crc	6.6 ± 0.8	5.8 ± 0.8	*p* < 0.001	6.2 ± 0.8	5.6 ± 0.9	*p* < 0.001
	R1/4	8.3 ± 0.9	7.2 ± 0.9	*p* < 0.001	7.4 ± 0.9	6.8 ± 0.9	*p* < 0.001
	R1/2	9.3 ± 1.2	8.5 ± 0.8	*p* < 0.001	9.0 ± 1.2	8.0 ± 0.9	*p* < 0.001
	R3/4	10.8 ± 1.0	10.2 ± 1.2	*p* = 0.0012	10.6 ± 1.0	9.7 ± 1.0	*p* < 0.001
	Rc	12.5 ± 1.1	11.6 ± 1.2	*p* < 0.001	12.1 ± 1.0	11.5 ± 1.2	*p* < 0.001

*SD* standard deviation, *Cr1/2* crown 1/2 complete, *Cr3/4* crown 3/4 complete, *Crc* crown complete, *R1/4* root length 1/4, *R1/2* root length 1/2, *R3/4* root length 3/4, *Rc* root length complete.

#### Lateral incisor

The data range for the upper lateral incisor was from Cr1/2 (1/2 crown formation) to Rc. The dental age of girls was significantly lower than that of boys in all developmental stages except Cr1/2 and R1/4. The data range for the lower lateral incisor was from Cr3/4 to Rc. The dental ages of Cr3/4, R1/2, and Rc were significantly lower in girls than in boys.

#### Canine

The data range for the upper canine was Cr1/2 to Rc. In all developmental stages, the dental age of girls was significantly lower than that of boys. The data range for the lower canine was Cr1/2 to Rc (as for the upper canine). There was no significant difference between boys and girls in Cr1/2, but girls showed significantly earlier growth than boys in all other developmental stages.

#### First premolar

The dental age by developmental stage of the premolars was calculated (Table 2). The data range for the upper first premolar was from Cco (coalescence of cusps) to Rc. The dental age of girls was significantly lower than that of boys for Cr3/4, R1/4, and R1/2, but there were no sex differences for the other developmental stages. The data range for the lower first premolar was from Cco to Rc (as for the upper first premolar). After Cr1/2 and Crc, at approximately 7 years of age, the dental age of girls was significantly lower than that of boys.

**Table 2:** Average age of premolar formation stages.

	Tooth stage	Upper			Lower		
		Male	Female	Sex difference	Male	Female	Sex difference
		Mean ± SD (years)	Mean ± SD (years)		Mean ± SD (years)	Mean ± SD (years)	
First premolar	Cco	3.9 ± 0.6	3.7 ± 0.4	*p* = 0.051	3.6 ± 0.2	3.7 ± 0.5	*p* = 0.54
	Cr1/2	4.8 ± 0.5	4.8 ± 0.7	*p* = 0.97	4.8 ± 0.6	4.4 ± 0.6	*p* < 0.001
	Cr3/4	6.2 ± 0.9	5.7 ± 0.7	*p* < 0.001	5.4 ± 0.7	5.4 ± 0.7	*p* = 0.70
	Crc	6.9 ± 0.8	6.8 ± 0.7	*p* = 0.65	7.0 ± 0.7	6.5 ± 0.8	*p* < 0.001
	R1/4	8.2 ± 0.7	7.8 ± 0.7	*p* = 0.0044	8.0 ± 0.9	7.5 ± 0.7	*p* < 0.001
	R1/2	9.3 ± 0.9	8.8 ± 0.8	*p* < 0.001	8.9 ± 1.0	8.5 ± 0.7	*p* = 0.026
	R3/4	10.3 ± 1.2	10.1 ± 1.1	*p* = 0.27	10.5 ± 1.1	10.1 ± 1.0	*p* = 0.013
	Rc	11.4 ± 1.0	11.1 ± 1.1	*p* = 0.064	11.8 ± 1.0	11.5 ± 0.9	*p* = 0.019
Second premolar	O	3.5 ± 0.3	3.6 ± 0.5	*p* = 0.85	–	3.5 ± 0.3	–
	Ci	4.0 ± 0.7	4.0 ± 0.6	*p* = 0.54	4.0 ± 0.6	4.0 ± 0.6	*p* = 0.66
	Cco	4.9 ± 0.6	5.1 ± 0.6	*p* = 0.10	4.7 ± 0.6	4.7 ± 0.7	*p* = 0.93
	Cr1/2	6.2 ± 0.8	5.8 ± 0.8	*p* = 0.0039	5.5 ± 0.7	5.4 ± 0.8	*p* = 0.66
	Cr3/4	6.8 ± 0.9	6.9 ± 0.9	*p* = 0.58	6.8 ± 0.8	6.4 ± 0.7	*p* = 0.0065
	Crc	8.0 ± 0.9	7.5 ± 0.7	*p* = 0.0030	7.7 ± 0.9	7.3 ± 0.7	*p* = 0.0092
	R1/4	8.9 ± 0.8	8.7 ± 0.8	*p* = 0.27	8.9 ± 1.0	8.3 ± 0.9	*p* = 0.0013
	R1/2	10.1 ± 1.2	9.8 ± 1.1	*p* = 0.052	10.0 ± 1.1	9.8 ± 1.1	*p* = 0.25
	R3/4	11.3 ± 1.1	11.0 ± 1.0	*p* = 0.048	11.2 ± 1.1	11.3 ± 1.3	*p* = 0.68
	Rc	12.4 ± 1.0	12.5 ± 1.2	*p* = 0.51	12.6 ± 1.1	12.3 ± 1.3	*p* = 0.063

*SD* standard deviation, *O* crypt, no calcification, *Cco* coalescence of cusps, *Ci* initial calcification, *Cr1/2* crown 1/2 complete, *Cr3/4* crown 3/4 complete, *Crc* crown complete, *R1/4* root length 1/4, *R1/2* root length 1/2, *R3/4* root length 3/4, *Rc* root length complete.

#### Second premolar

For the upper second premolar, data were available for all developmental stages from O to Rc. The dental age of Cr1/2, Crc, and R3/4 was significantly lower in girls; however, there were no sex differences in the other developmental stages. The data range for the lower second premolar was all developmental stages from O to Rc for girls (as for the upper second premolar), but was Ci (initial calcification) to Rc for boys. The dental ages of Cr3/4, Crc, and R1/4, from approximately 6.5 to 8.5 years of age, were significantly earlier in girls, but no sex differences were observed in the other developmental stages.

#### First molar

The dental age by developmental stage of the molars was calculated (Table 3). The data range for the upper first molar was from Cr3/4 to Rc. No difference was observed between the sexes until Crc at approximately 4.5 years of age, but girls were significantly younger at after R1/4 at approximately 5 years of age. The data range for the lower first molar was Cr3/4 to Rc for boys (as for the upper first molar), but Crc to Rc for girls. No sex difference was observed in the dental age of R1/2, but the average age of girls was significantly lower in all other developmental stages.

**Table 3:** Average age of molar formation stages.

	Tooth stage	Upper			Lower		
		Male	Female	Sex difference	Male	Female	Sex difference
		Mean ± SD (years)	Mean ± SD (years)		Mean ± SD (years)	Mean ± SD (years)	
First molar	Cr3/4	3.7 ± 0.5	3.7 ± 0.5	*p* = 0.89	3.5 ± 0.3	–	–
	Crc	4.6 ± 0.7	4.5 ± 0.7	*p* = 0.48	3.9 ± 0.5	3.7 ± 0.5	*p* = 0.019
	R1/4	5.5 ± 0.8	5.2 ± 0.7	*p* = 0.013	4.9 ± 0.6	4.6 ± 0.5	*p* = 0.0012
	R1/2	6.3 ± 0.7	5.7 ± 0.8	*p* < 0.001	5.6 ± 0.8	5.5 ± 0.6	*p* = 0.28
	R3/4	7.2 ± 0.8	6.8 ± 0.9	*p* = 0.042	6.7 ± 0.8	6.3 ± 0.7	*p* = 0.0020
	Rc	8.5 ± 1.1	7.8 ± 1.0	*p* < 0.001	8.3 ± 1.0	7.8 ± 1.0	*p* < 0.001
Second molar	O	3.8 ± 0.4	3.8 ± 0.6	*p* = 0.72	3.6 ± 0.3	3.6 ± 0.4	*p* = 0.86
	Ci	4.2 ± 0.6	4.4 ± 0.7	*p* = 0.20	4.4 ± 0.7	4.3 ± 0.7	*p* = 0.42
	Cco	5.1 ± 0.8	5.3 ± 1.1	*p* = 0.48	5.2 ± 0.7	5.1 ± 0.8	*p* = 0.44
	Cr1/2	6.0 ± 0.8	6.1 ± 0.7	*p* = 0.42	6.2 ± 1.0	6.2 ± 0.8	*p* = 0.99
	Cr3/4	7.3 ± 0.8	7.0 ± 0.8	*p* = 0.043	7.4 ± 0.8	7.1 ± 0.8	*p* = 0.026
	Crc	8.5 ± 1.0	8.2 ± 0.9	*p* = 0.049	8.6 ± 0.8	8.1 ± 0.9	*p* < 0.001
	R1/4	9.9 ± 0.9	9.6 ± 1.1	*p* = 0.062	9.5 ± 1.1	9.4 ± 1.0	*p* = 0.45
	R1/2	11.1 ± 1.0	10.8 ± 0.9	*p* = 0.082	10.6 ± 1.0	10.4 ± 0.9	*p* = 0.26
	R3/4	12.1 ± 1.1	12.0 ± 1.4	*p* = 0.56	11.6 ± 1.0	11.7 ± 1.1	*p* = 0.55
	Rc	13.8 ± 1.2	13.6 ± 1.4	*p* = 0.20	13.4 ± 1.1	13.3 ± 1.4	*p* = 0.60
Third molar	O	9.8 ± 1.5	10.3 ± 1.2	*p* = 0.32	10.0 ± 2.0	9.9 ± 1.4	*p* = 0.15
	Ci	10.4 ± 1.4	10.5 ± 1.7	*p* = 0.96	10.7 ± 1.3	10.5 ± 1.3	*p* = 0.69
	Cco	11.3 ± 1.4	10.8 ± 1.5	*p* = 0.11	11.6 ± 1.8	11.6 ± 2.0	*p* = 0.47
	Cr1/2	12.2 ± 1.4	12.1 ± 1.7	*p* = 0.79	13.1 ± 1.6	13.3 ± 1.9	*p* = 0.83
	Cr3/4	13.3 ± 1.3	13.6 ± 1.6	*p* = 0.33	14.0 ± 1.6	14.0 ± 1.7	*p* = 0.50
	Crc	14.9 ± 1.5	14.3 ± 1.8	*p* = 0.038	14.9 ± 1.2	15.6 ± 1.6	*p* = 0.89
	R1/4	15.7 ± 1.4	16.3 ± 1.6	*p* = 0.044	15.8 ± 1.1	16.2 ± 1.4	*p* = 0.012
	R1/2	16.7 ± 1.2	16.6 ± 1.4	*p* = 0.76	16.4 ± 1.3	16.9 ± 1.5	*p* = 0.19
	R3/4	16.8 ± 0.9	17.2 ± 1.2	*p* = 0.17	17.3 ± 1.1	–	–
	Rc	17.8 ± 0.8	–	–	17.5 ± 0.9	–	–

*SD* standard deviation, *O* crypt, no calcification, *Cco* coalescence of cusps, *Ci* initial calcification, *Cr1/2* crown 1/2 complete, *Cr3/4* crown 3/4 complete, *Crc* crown complete, *R1/4* root length 1/4, *R1/2* root length 1/2, *R3/4* root length 3/4, *Rc* root length complete.

#### Second molar

For the upper second molar, data were available for all developmental stages from O to Rc. The dental age of Cr3/4 and Crc, from approximately 7 to 8.5 years, was significantly lower in girls, but there were no sex differences in other developmental stages. For the lower second molar, data were also available for all developmental stages from O to Rc. The dental age of girls was significantly lower for Cr3/4 and Crc, at approximately 7 to 8.5 years of age, but there were no sex differences in other developmental stages.

#### Third molar

The data range for the upper third molar was all developmental stages from O to Rc for boys, but O to R3/4 for girls. Crc, at approximately 14.5 years of age, was significantly earlier in girls, but R1/4, at approximately 16 years of age, was significantly earlier in boys. The data range for the lower third molar was all developmental stages from O to Rc for boys (as for the upper third molar), but O to R1/2 for girls. Crc was significantly earlier in boys. The standard deviation ranged from 0.8 to 2.0 years in boys and from 1.2 to 2.0 years in girls, indicating that the greatest variability in the sample data was for permanent teeth.

### Comparison of time required for root formation

The time required for root formation was compared using the interval between Crc and Rc (Table 4). The root formation time was slowest for the canine, with approximately 6 years from Crc to Rc. In boys, the permanent tooth with the fastest root formation rate was the third molar, with approximately 2.5 to 3 years from Crc to Rc. In girls, there were no Rc data for the third molar, so the rate of root formation could not be determined.

**Table 4:** Order of time required from crown completion to root length completion.

Order	Upper				Lower			
	Male		Female		Male		Female	
	Tooth type	Required time (years)	Tooth type	Required time (years)	Tooth type	Required time (years)	Tooth type	Required time (years)
1	Third molar	2.9	First molar	3.3	Third molar	2.6	Lateral incisor	3.3
2	Central incisor	3.6	Central incisor	3.4	Central incisor	3.3	Central incisor	3.4
3	Lateral incisor	3.7	Lateral incisor	3.7	Lateral incisor	3.6	First molar	4.1
4	First molar	3.9	First premolar	4.3	First molar	4.4	First premolar	5.0
5	Second premolar	4.4	Second premolar	5.0	First premolar	4.8	Second premolar	5.0
6	First premolar	4.5	Second molar	5.4	Second molar	4.8	Second molar	5.2
7	Second molar	5.3	Canine	5.8	Second premolar	4.9	Canine	5.9
8	Canine	5.9			Canine	5.9		

### Calculation of 50th percentile developmental stage of permanent teeth at each age

#### Central incisor

The 50th percentile developmental stage of the anterior teeth at each age was calculated (Table 5). The upper central incisor reached Crc at 4 years in both sexes. The upper central incisor reached Ac (apex closed) at 10 years in boys and 9 years in girls. The lower central incisor reached Ac at 8 years in both sexes.

**Table 5:** Median of developmental stage for each tooth according to age of anterior teeth.

Age (years)	Central incisor				Lateral incisor				Canine			
	Male		Female		Male		Female		Male		Female	
	Upper	Lower	Upper	Lower	Upper	Lower	Upper	Lower	Upper	Lower	Upper	Lower
3	Cr3/4	Cr3/4	Cr3/4	Cr3/4	Cr1/2	Cr3/4	Cr1/2	Cr3/4	Cr1/2	Cr1/2	Cr1/2	Cr1/2
4	Crc	Crc	Crc	Crc	Cr3/4	Cr3/4	Cr3/4	Crc	Cr3/4	Cr3/4	Cr3/4	Cr3/4
5	Crc	R1/4	Crc	R1/4 R1/2	Cr3/4	Crc	Crc	Crc R1/4	Cr3/4	Crc	Crc	Crc
6	R1/4	R1/2 R3/4	R1/2	R3/4	R1/4	R1/2	R1/4	R1/2	Crc	Crc	Crc R1/4	R1/4
7	R1/2	Rc	R3/4	Rc	R1/4	R3/4	R1/2	R3/4	Crc R1/4	R1/4	R1/4	R1/4
8	R3/4	Ac	Rc	Ac	R3/4	Rc	R3/4	Rc	R1/4	R1/2	R1/2	R1/2
9	Rc	Ac	Ac	Ac	Rc	Ac	Rc Ac	Ac	R1/2	R1/2	R1/2	R3/4
10	Ac	Ac	Ac	Ac	Ac	Ac	Ac	Ac	R3/4	R3/4	R3/4	Rc
11	Ac	Ac	Ac	Ac	Ac	Ac	Ac	Ac	Rc	Rc	Rc	Rc
12	Ac	Ac	Ac	Ac	Ac	Ac	Ac	Ac	Rc	Rc	Ac	Ac
13	Ac	Ac	Ac	Ac	Ac	Ac	Ac	Ac	Ac	Ac	Ac	Ac
14	Ac	Ac	Ac	Ac	Ac	Ac	Ac	Ac	Ac	Ac	Ac	Ac
15	Ac	Ac	Ac	Ac	Ac	Ac	Ac	Ac	Ac	Ac	Ac	Ac
16	Ac	Ac	Ac	Ac	Ac	Ac	Ac	Ac	Ac	Ac	Ac	Ac
17	Ac	Ac	Ac	Ac	Ac	Ac	Ac	Ac	Ac	Ac	Ac	Ac
18	Ac	Ac	Ac	Ac	Ac	Ac	Ac	Ac	Ac	Ac	Ac	Ac

*Cr1/2* crown 1/2 complete, *Cr3/4* crown 3/4 complete, *Crc* crown complete, *R1/4* root length 1/4, *R1/2* root length 1/2, *R3/4* root length 3/4, *Rc* root length complete, *Ac* apex closed.

#### Lateral incisor

The upper lateral incisor passed Crc at 5 to 6 years, and the lower lateral incisor reached Crc at 5 years in boys. The Ac age was 10 years for the upper lateral incisor and 9 years for the lower lateral incisor. In girls, the upper lateral incisor reached Crc at 5 years and the lower lateral incisor reached Crc at 4 years. In contrast, the Ac age was 9 years for both upper and lower lateral incisors.

#### Canine

The upper canine reached Crc at 6 years in boys. The lower canine in boys and the upper and lower canines in girls reached Crc at 5 years. Ac was reached in the upper and lower canines of both sexes at 13 and 12 years, respectively. In addition, the central incisor, lateral incisor, and canine reached Crc at similar ages, but Ac was delayed in the canine by more than 2 years.

#### First premolar

The 50th percentile developmental stage of the premolars at each age was calculated (Table 6). The upper and lower first premolars of both sexes reached Crc at 6 years. Ac was reached in the upper and lower first premolars of both sexes at 13 and 12 years, respectively.

**Table 6:** Median developmental stage in each tooth according to age of premolars.

Age (years)	First premolar				Second premolar			
	Male		Female		Male		Female	
	Upper	Lower	Upper	Lower	Upper	Lower	Upper	Lower
3	Cco	Cco	Cco	Cco	O Ci	Ci	O	O
4	Cr1/2	Cr1/2	Cr1/2	Cr1/2	Cco	Cco	Cco	Cco
5	Cr1/2	Cr3/4	Cr3/4	Cr3/4	Cr1/2	Cr1/2	Cr1/2	Cr1/2
6	Crc	Crc	Crc	Crc	Cr3/4	Cr3/4	Cr3/4	Cr3/4
7	Crc	Crc	R1/4	R1/4	Cr3/4	Crc	Crc	Crc
8	R1/4	R1/4	R1/2	R1/2	Crc	R1/4	R1/4	R1/4
9	R1/2	R3/4	R1/2	R3/4	R1/2	R1/2	R1/2	R1/2
10	R3/4	R3/4	Rc	R3/4	R1/2	R1/2	R3/4	R1/2 R3/4
11	Rc	Rc	Rc	Rc	R3/4	R3/4	Rc	R3/4
12	Rc	Rc	Ac	Ac	Rc	Rc	Rc	Rc
13	Ac	Ac	Ac	Ac	Ac	Ac	Ac	Ac
14	Ac	Ac	Ac	Ac	Ac	Ac	Ac	Ac
15	Ac	Ac	Ac	Ac	Ac	Ac	Ac	Ac
16	Ac	Ac	Ac	Ac	Ac	Ac	Ac	Ac
17	Ac	Ac	Ac	Ac	Ac	Ac	Ac	Ac
18	Ac	Ac	Ac	Ac	Ac	Ac	Ac	Ac

*O* crypt, no calcification, *Cco* coalescence of cusps, *Ci* initial calcification, *Cr1/2* crown 1/2 complete, *Cr3/4* crown 3/4 complete, *Crc* crown complete, *R1/4* root length 1/4, *R1/2* root length 1/2, *R3/4* root length 3/4, *Rc* root length complete, *Ac* apex closed.

#### Second premolar

In boys, the upper second premolar and lower second premolar reached Crc at 8 and 7 years, respectively. In girls, the upper and lower second premolars reached Crc at 7 years. In girls, Ac was reached in the upper second premolar at 12 years and the lower second premolar at 13 years. In boys, Ac was reached in the upper and lower second premolars at 13 years.

#### First molar

The 50th percentile developmental stage of the molars at each age was calculated (Table 7). For both sexes, the upper first molar reached Crc at 4 years and the lower first molar at 3 years. In both sexes, the upper and lower first molars reached Ac at 9 years.

**Table 7:** Median of developmental stage for each tooth according to age of molars.

Age (years)	First molar				Second molar				Third molar			
	Male		Female		Male		Female		Male		Female	
	Upper	Lower	Upper	Lower	Upper	Lower	Upper	Lower	Upper	Lower	Upper	Lower
3	Cr3/4	Cr3/4 Crc	Cr3/4	Crc	O	O	O	O	NS	NS	NS	NS
4	Crc	R1/4	Crc	R1/4	Cco	Cco	Ci	Cco	NS	NS	NS	NS
5	R1/4	R1/2	R1/4	R1/2	Cr1/2	Cco	Cco	Cco	NS	NS	NS	NS
6	R1/2	R3/4	R3/4	R3/4	Cr1/2	Cr1/2	Cr1/2	Cr1/2 Cr3/4	NS	NS	NS	NS
7	R3/4	Rc	Rc	Rc	Cr3/4	Cr3/4	Cr3/4	Cr3/4	NS	NS	NS	NS
8	Rc	Rc	Rc	Rc	Crc	Crc	Crc	Crc	NS	NS	NS	NS
9	Ac	Ac	Ac	Ac	R1/4	R1/4	R1/4	R1/4	NS	O	NS	O
10	Ac	Ac	Ac	Ac	R1/2	R1/2	R1/2	R1/2	Ci	Ci	Ci	Ci
11	Ac	Ac	Ac	Ac	R1/2	R3/4	R3/4	R3/4	Cco	Cco	Cco Cr1/2	Cco
12	Ac	Ac	Ac	Ac	R3/4	Rc	Rc	Rc	Cr1/2	Cco	Cr1/2	C1/2
13	Ac	Ac	Ac	Ac	Rc	Rc	Rc	Rc	Cr3/4	Cr1/2	Cr3/4	Cr3/4
14	Ac	Ac	Ac	Ac	Rc	Rc Ac	Ac	Ac	Crc	Cr3/4	Cr3/4	Cr3/4
15	Ac	Ac	Ac	Ac	Ac	Ac	Ac	Ac	R1/4	R1/4	R1/4	Crc
16	Ac	Ac	Ac	Ac	Ac	Ac	Ac	Ac	R1/4	R1/4	Crc	Crc R1/4
17	Ac	Ac	Ac	Ac	Ac	Ac	Ac	Ac	R3/4	R3/4	R1/2	R1/2
18	Ac	Ac	Ac	Ac	Ac	Ac	Ac	Ac	Rc	Rc	R3/4	R1/2

*NS* not significant, *O* crypt, no calcification, *Cco* coalescence of cusps, *Ci* initial calcification, *Cr1/2* crown 1/2 complete, *Cr3/4* crown 3/4 complete, *Crc* crown complete, *R1/4* root length 1/4, *R1/2* root length 1/2, *R3/4* root length 3/4, *Rc* root length complete, *Ac* apex closed.

#### Second molar

The upper and lower second molars of both sexes reached Crc at 8 years. Ac was reached in the upper and lower second molars of girls, and the lower second molar of boys, at 14 years. Ac was reached in the upper second molar of boys at 15 years.

#### Third molar

In boys, the upper third molar reached Crc at 14 years, and the lower third molar passed Crc at 14–15 years. In girls, the upper third molar reached Crc at 16 years and the lower third molar reached Crc at 15 years. In both sexes, less than half of the third molars reached Ac at age 18 years, the upper limit of the target age range.

### Reliability of the study model

#### Correlation between total and variance of developmental scores

Cronbach’s alpha coefficients^20^ of 0.987 for boys and 0.986 for girls were both above 0.8, which shows high internal consistency in the sample data (Supplementary Table 1). This indicates that the evaluation criteria for the developmental stages of permanent teeth used by the raters in this study were not affected by the passage of time.

#### Confirmation by different evaluators

The Kendall’s rank correlation coefficient^21^ for the association between total dental age and chronological age in the test data was τ = 0.805, which is above 0.8, indicating a very strong correlation. This suggested that the method of evaluating the developmental stages of permanent teeth and the data obtained in this study were highly versatile, even when different evaluators were used.

## Discussion

Orthopantomography is a method of visualizing the jawbone and teeth in a curved fault zone^22^ and can be used to diagnose the formation stage of unerupted teeth. Mattila and Haavikko^9^ demonstrated a close correspondence between clinical and orthopantomographic findings for first molars, and reported that the data were highly accurate. A longitudinal study by Fanning^23^ investigated the relationship between permanent tooth development and chronological age using orthopantomography. However, this method exposes the examinee to additional radiation and is therefore problematic from a modern ethical perspective. In contrast, Haavikko^8^ conducted a cross-sectional study using orthopantomograph samples collected in a medically safe manner that did not involve additional radiation exposure. In this cross-sectional study, we safely obtained a large 10-year sample (2009–2019) of orthopantomographs of Japanese individuals, which allowed us to create a parametric-equivalent sample and perform various tests to obtain reliable data. As a result, we were able to develop a method for assessing dental age that is appropriate for modern Japanese children and adolescents.

The embryogenesis of the central incisor begins at approximately 4 to 5 months of fetal age, the first molar in the neonatal period, the canine at approximately 1 to 6 months of age, and the lateral incisor at approximately 10 to 11 months of age^13^. In addition, the formation of the first premolar begins at approximately 1.8 years of age^24^. The crown formation of these permanent teeth had already progressed at 3 years, which was the lower limit of the target age range in this study, and several developmental stages could not be calculated. However, because orthopantomography is used for oral examinations in pediatric dentistry after the age of 3 years, when children can receive dental treatment, data obtained before the age of 3 years are of little importance. We found that the third molar had not yet reached the Ac stage at 18 years, which was the upper age limit for this study. Therefore, additional data would be useful using a wider target age range.

Using orthopantomography, Hirano et al.^25^ identified a right–left difference in the growth rate of the first molars in up to 12.4% of cases of children aged approximately 6 years. In the present study, we investigated the occurrence of left–right differences in dental age at different developmental stages. The results showed that there was no significant difference in the growth rates of bilateral homonymous teeth at any developmental stage. This suggests that contralateral homonymous teeth can be used as a reference when it is difficult to identify the developmental stage of a tooth owing to tooth inclination problems/anomalies or lack of orthopantomograph clarity.

We found that girls tended to develop permanent teeth earlier than boys, but there were fewer significant differences in developmental stages of the second premolar, second molar, and third molar, which reached the Rc stage after 12 years of age. Furthermore, at 15 years, the age at which secondary male sexual characteristics develop, boys showed significantly earlier development of the third molar than girls. These results suggest that the development of permanent teeth corresponds to the general type as well as the jawbone of Scammon’s development curve, and that girls develop faster than boys until puberty. Boys then catch up with girls after puberty owing to their rapid development.

All permanent teeth, except the third molar, had reached Rc by the age of 13.8 years for boys and 13.6 years for girls. A study conducted approximately 50 years ago in Finland reported that all permanent teeth reached Rc by 16.2 years in boys and 15.1 years in girls^8^, and a study conducted 5 years ago in Iceland showed that all permanent teeth had reached Rc by 13.8 years in boys and 13.3 years in girls^26^. In comparison with these earlier findings, the present data show that Japanese children and adolescents reach Rc earlier than Finns (older data), but at approximately the same age as Icelanders (more recent data). This suggests that separate conversion table of dental age of permanent teeth by developmental stage are needed for each population, and that these should be regularly updated.

The rate of root formation was clarified in this study. The root length of the upper canine was 14.5 mm and that of the lower canine was 13.6 mm^27^, suggesting that the rate of root formation is slower for longer root lengths. However, the root length of the third molar was 9.5 mm for the upper molar and 9.9 mm for the lower molar; in boys, these teeth showed the shortest root length and the fastest root formation rate. Although there were no third molar data for girls, the upper central incisor, upper first molar, lower central incisor, and lower lateral incisor related to Hellman’s tooth age stage IIIA reached complete root formation in a short period of time, suggesting that eruption time also affects the root formation rate.

The treatment of the third molar in the calculation of dental age has been the subject of various debates^28,29^. Kullman et al.^30^ attempted to establish a method of estimating chronological age from the developmental stage of the third molar using a computer program with intraoral radiograph data, but the results were unreliable. Our results also showed that the standard deviation of the dental age of the third molar by developmental stage was remarkably large, ranging from 0.8 to 2.0 years. Dental age is considered as important as carpal bone ossification age in estimating age under 20 years in forensic medicine^31^; therefore, we considered that the third molar, which is the last to reach complete root formation, is the best indicator, although it is less accurate than other methods.

When building a model for unknown data, such as the present conversion table of dental age of permanent teeth by developmental stage, it is necessary to examine its applicability to the population by examining whether there is any bias toward a particular result. We were able to confirm the reliability and generalizability of our results by examining the differences between the sample raters’ evaluation criteria over time and the test data from different raters. This showed that the present findings on the developmental stages of permanent teeth could be generalized to the whole population of modern Japanese children and adolescents. Our data were obtained from orthopantomographs, which are often used for orthodontic evaluation in potential cases of problems in dental, oral, and craniofacial development. Therefore, these results may not be representative of the healthy population. However, if used in combination with data on the eruption time of permanent teeth^32^, our method could improve the accuracy of dental age assessment.

In this study, a dental maturity index for children under 18 years of age was determined by establishing a method for calculating dental age in modern Japanese individuals. The use of this dental age calculation method in clinical pediatric dentistry would enable accurate assessment of permanent tooth growth and lead to appropriate diagnosis and treatment decisions. However, in late adolescence (after the age of 14 years), when the development of almost all permanent teeth is complete, the number of permanent teeth that can be used to calculate dental age declines, reducing the reliability of the results. This problem could be resolved if data on the dental age of Ac of each permanent tooth and the third molar could be obtained. This would further expand the applicable age range of this dental age calculation method.

In summary, a large-scale analysis of the latest orthopantomographs was conducted to investigate the dental age of permanent teeth in the Japanese population. The following results were obtained. The dental age of each permanent tooth from infancy to mid-adolescence was clarified, and a method established for calculating the total dental age suitable for modern Japanese individuals. In modern Japanese children and adolescents, girls tend to develop at a faster rate than boys, but boys tend to catch up with girls owing to secondary sexual characteristics. We developed a method that could be used to evaluate the correct dental age for modern Japanese children and adolescents, and clarified the current status of the relationship between the rate of development of permanent teeth and chronological age.

## Material and methods

### Collection of orthopantomographs

#### Sample and screening conditions

In this retrospective study, we used orthopantomographs stored in the electronic media database of the target dental facilities: Osaka University Dental Hospital, Kuremoto General and Pediatric Dentistry, Team White Nishikawa Dental Clinic, Tokiwa-kai Kuremoto Dental Clinic, and Tokiwa-kai Kuremoto Dental Clinic in Namba. Of the orthopantomographs obtained over a 10-year period (2009 to 2019), we used those from individuals aged from 3 years old, when orthopantomography for diagnosis can first be performed, to 18 years old, when height growth is almost complete. Our sample contained only orthopantomograms from Japanese nationals.

The main reasons for clinical attendance for patients in the sample were dental caries, trauma treatment, and dental checkups. We excluded orthopantomographs of individuals with systemic diseases that may affect the number, morphology, and growth rate of permanent teeth owing to physical events. We also excluded orthopantomographs of individuals with cleft lip and palate, supernumerary teeth, eruption disturbances (except for permanent third molars), delayed of eruption, dental agenesis, congenitally missing teeth, and teeth with a history of endodontic treatment.

#### Determination of sample size

To eliminate bias in the number of orthopantomographs by chronological age, the sample was defined as 16 orthopantomography age groups of 3 to 18 years, divided into 1-year intervals. An equal number of images were collected from each group for boys and girls at each age (the sample proportion for each group was 6.25%). On the assumption that the population was normally distributed, the 95% confidence interval of the population ratio was obtained using the following equation.

$${\text{r}} - 1.96 \times \sqrt {\frac{{ {\text{r}} \left( { 1 - {\text{r}} } \right)}}{n}} \le p \le {\text{r}} + 1.96 \times \sqrt {\frac{{ {\text{r}} \left( { 1 - {\text{r}} } \right)}}{n}}$$

To set the confidence coefficient at 95% and the confidence interval for the sample proportion (0.0625) at less than 5%, the following relationship was established.

$$2 \, \times \,1.96 \, \times \,\sqrt {\frac{{ 0.0625\, \left( { 1 - 0.0625 } \right)}}{n}} \le 0.05$$

The sample size calculated from this equation was a minimum of 361 images. If the number of orthopantomographs for each age was set to 24 (12 for each sex), a minimum of 384 images would be required. Furthermore, in accordance with the central limit theorem^33^, the number of orthopantomographs for each age was set to 64 (32 for each sex) for a sample size of 1024, to bring the population closer to a normal distribution by increasing the sample size.

#### Evaluation method for dental age

**Figure 1:**
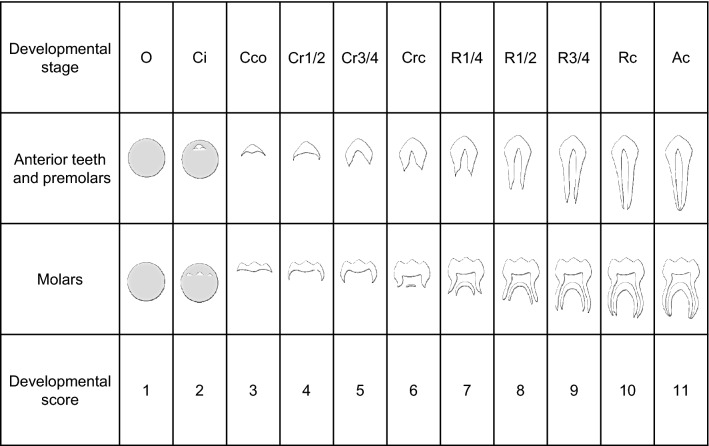
Developmental stages of permanent teeth. Stages of crown formation (O: crypt, no calcification; Ci: initial calcification; Cco: coalescence of cusps; Cr1/2: crown 1/2 complete; Cr3/4: crown 3/4 complete; Crc: crown complete). Stages of root formation (R1/4: root length 1/4; R1/2: root length 1/2; R3/4: root length 3/4; Rc: root length complete; Ac: apex closed).

Based on Haavikko’s classification of tooth developmental stages, 32 permanent tooth types on the left and right sides of the upper and lower jaws were categorized into two groups: a group of anterior teeth and premolars, and a group of molars. Each developmental stage was classified into 11 stages (Fig. 1). In accordance with this classification, the developmental stages of 32 permanent tooth types on the right and left sides of the upper and lower jaws were evaluated using orthopantomography. A tooth that had passed the beginning of any given stage was regarded as belonging to the formation stage until the tooth reached the beginning of the next stage.

In the evaluation of the root formation stage of the molar group, the crown-to-root ratio was taken into account (in addition to the evaluation criteria shown in Fig. 1) owing to the diversity in the number of roots and the presence of root morphological abnormalities such as prism-shaped root and gutter-shaped root. The crown length of the upper and lower first and second molars is 7.0 mm to 7.9 mm, and the root length is 11.0 mm to 12.0 mm^27^. Therefore, when the crown length is 1.0, the ratio of root length less than 0.5 was defined as R1/4, 0.5 to 1.0 was defined as R1/2, 1.0 to 1.5 was defined as R3/4, and a ratio exceeding 1.5 was defined as Rc. The third molar was evaluated using the same criteria. In the event of differences in the formation of each root (e.g., if the formation of the mesial root was early and the formation of the other roots was late), the stage of the root with the slowest formation was used.

All samples were evaluated by one dentist (although two other dentists evaluated the samples for the reliability analysis, as described in “Discussion” section). The process of evaluating 1024 orthopantomographs began in April 2017 and was completed by April 2020. Each of the 11 stages of permanent tooth development was scored using a numerical value ranging from 1 to 11 points and used as the developmental score in each survey.

### Statistical analysis

#### Significant difference in bilateral homonymous teeth

Microsoft Excel® (Microsoft Corporation, Washington, USA) was used for data tabulation and statistical processing. The difference between the right and left sides was tested to investigate the distribution of age at each developmental stage in the bilateral homonymous teeth of boys and girls. Student’s t-test was used; *p* < 0.05 was considered to indicate statistical significance.

#### Survey of chronological age distribution at each developmental stage

Cumulative frequency distribution charts are linear if the data are close to a uniform distribution and show a sigmoid curve if the data are close to a normal distribution. Therefore, to investigate the distribution of the study data, cumulative frequency distribution charts for age at each developmental stage of permanent teeth were generated, and normality was visually confirmed from the waveforms. The only developmental stages of permanent teeth shown in the cumulative frequency distributions are Crc and Rc; the results were considered applicable to all developmental stages.

#### Calculation of average age by tooth developmental stage

The developmental stages of permanent teeth evaluated using 1024 orthopantomographs were tabulated by sex and age, and the dental age and data variability for each developmental stage were calculated. The results were expressed in decimal notation following Haavikko^8^. Subsequently, Student’s t-test was used to examine sex differences in dental age by developmental stage; *p* < 0.05 was considered to indicate statistical significance. Because the upper limit of the target age for Ac is unknown and it is difficult to calculate the dental age, it was excluded from the results.

#### Comparison of the developmental order of permanent teeth

The developmental order of each permanent tooth was compared, focusing on the dental age of Crc and Rc, which are considered important landmarks in the development of permanent teeth between O and Ac.

#### Calculation of tooth developmental stages at each chronological age divided into 1-year intervals

We calculated the 50th percentile developmental stages of permanent teeth at each age from 3 to 18 years. The 50th percentile developmental stage from 3 years (the lower limit of the target range) to 18 years (the upper limit of the target range) was specified as the data range for each permanent tooth in the conversion table of dental age by tooth developmental stage. In addition, a comparative study was conducted focusing on the age at which each permanent tooth reached Rc and Ac.

### Reliability of the study model

#### Correlation between total tooth developmental scores and variance

Cronbach’s alpha expresses the internal consistency or average correlation in a study and is a measure of reliability^20^. In this study, it took 3 years to complete the survey of 1024 orthopantomographs, and we were concerned that the evaluators’ criteria for the developmental stage of permanent teeth may have changed over time. We calculated Cronbach’s alpha coefficients for boys and girls using the developmental scores to examine the internal consistency of the sample data. The data for the bilateral homonymous teeth were considered approximate and may have affected the results, so they were calculated as separate samples.

#### Confirmation of reliability by different evaluators

As test data, 44 new orthopantomographs (26 boys and 18 girls) from individuals aged 3 to 18 years were collected, and two dentists evaluated the developmental stages of permanent teeth. Using the conversion table of dental age by tooth developmental stage generated in this study, we calculated the respective total dental age of each individual. Correlations between dental age and chronological age for all permanent teeth were used to confirm the general applicability of the conversion table of dental age for the evaluation of each stage of permanent tooth development obtained from the two evaluators. Kendall’s rank correlation coefficient^21^ was used for this calculation.

### Ethical considerations

This study fully adhered to the principles of the Declaration of Helsinki (64th World Medical Association General Assembly, Fortaleza, Brazil, 2013), and the study protocols were approved by the ethics committee of Osaka University Graduate School of Dentistry (approval no. H29-E4). All data were fully anonymized before they were accessed for this study, and the ethics committee of Osaka University Graduate School of Dentistry waived the requirement to obtain informed consent from patients. Because this was a retrospective observational study using only existing medical records, informed consent was obtained via opt-out on our hospital website. Patients who opted out were excluded from the study.

## Supplementary Information

Supplementary Information 1.

Supplementary Information 2.

Supplementary Information 3.

Supplementary Information 4.

## Data Availability

The original contributions presented in the study are included in the article; further inquiries can be directed to the corresponding author.
