# Supplementary material for: Estimation of dental age based on the developmental stages of permanent teeth in Japanese children and adolescents
Source: Sci Rep. 2022 Feb 28;12:3345. doi: 10.1038/s41598-022-07304-2 (PMC8885679; doi:10.1038/s41598-022-07304-2)
Supplement: Supplementary file 1 — Supplementary Information 1. [file 41598_2022_7304_MOESM1_ESM.pptx]

## Slide 1
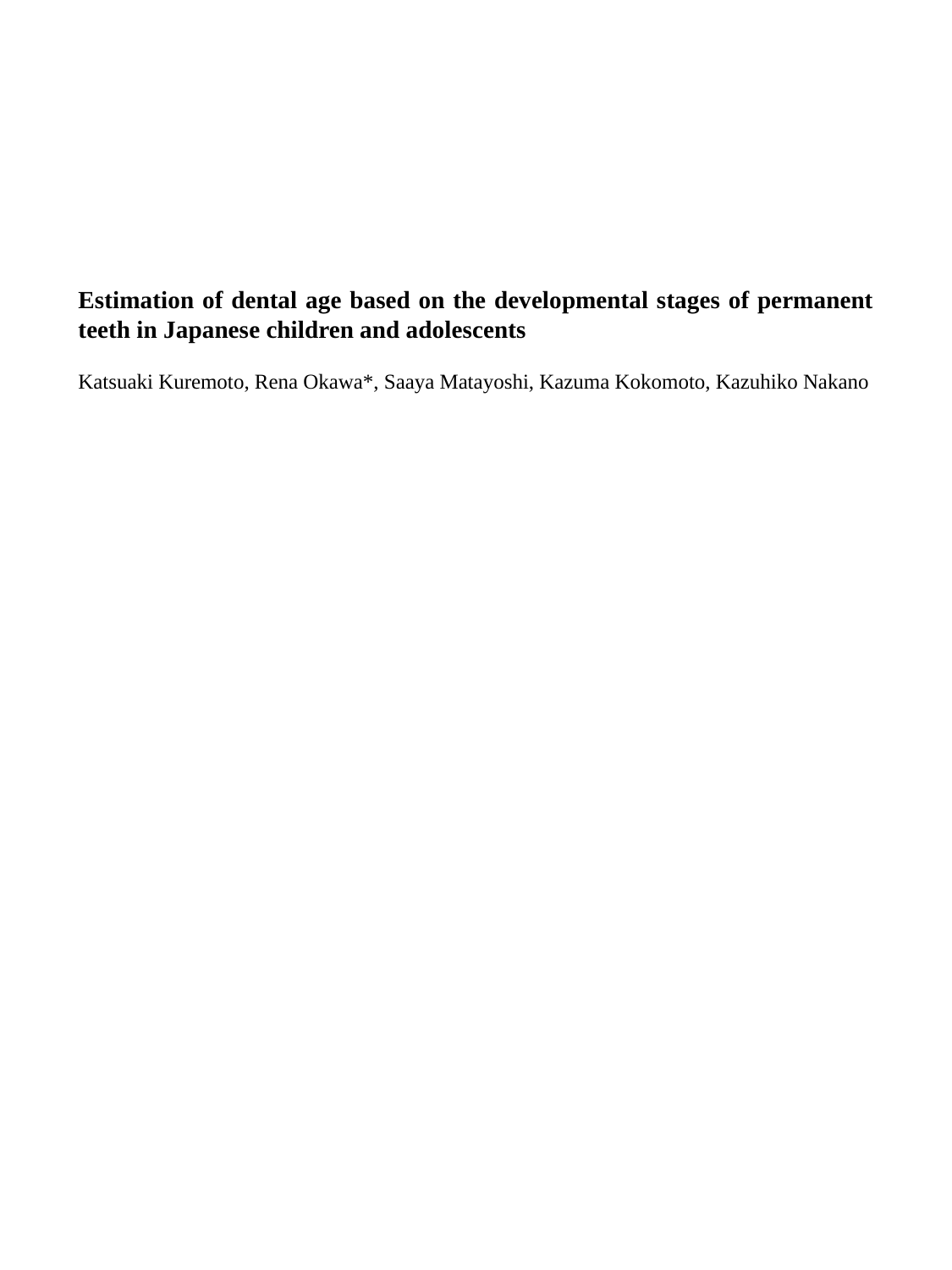

Estimation of dental age based on the developmental stages of permanent teeth in Japanese children and adolescents
Katsuaki Kuremoto, Rena Okawa*, Saaya Matayoshi, Kazuma Kokomoto, Kazuhiko Nakano

## Slide 2
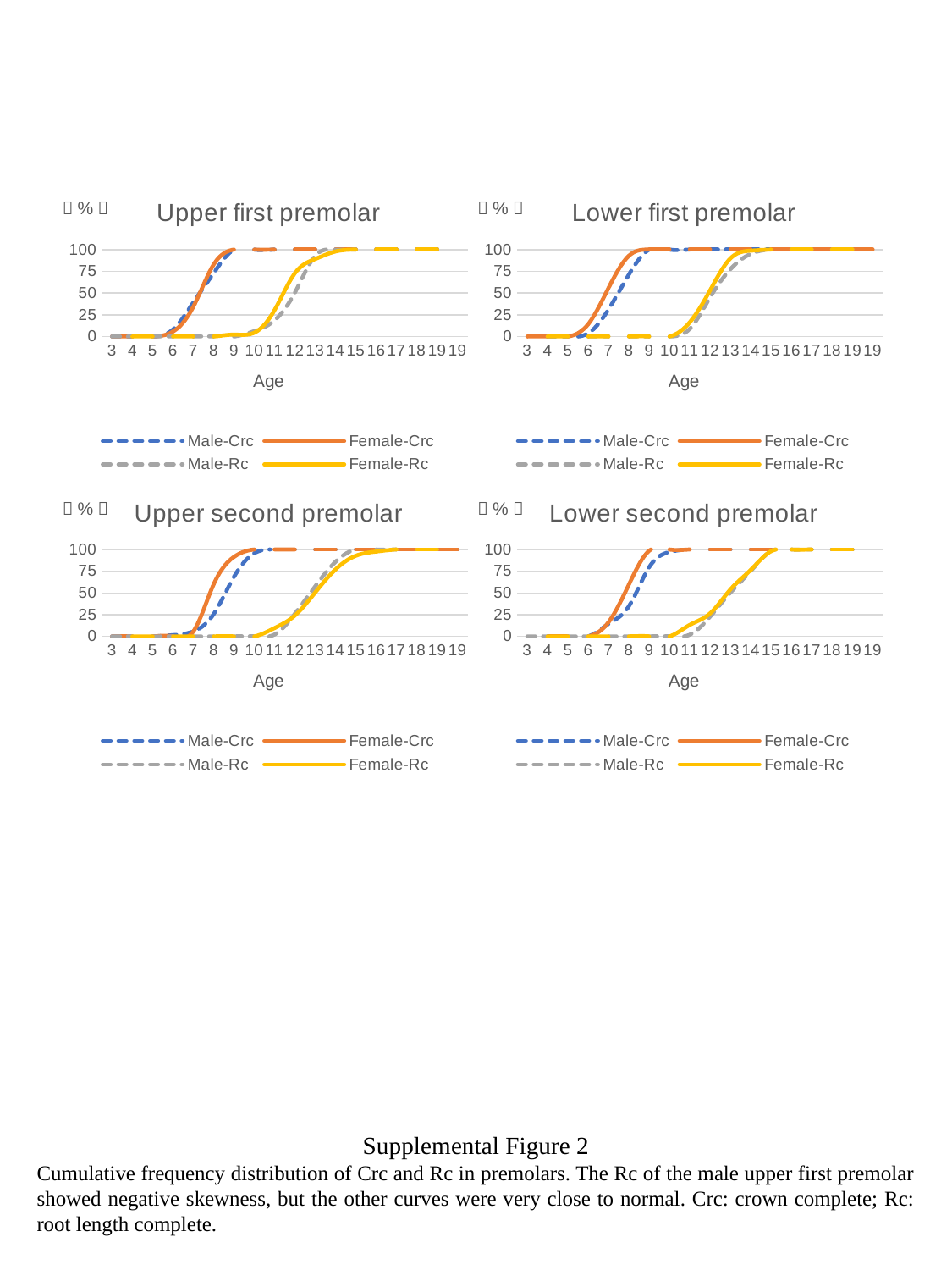

### Chart: Upper first premolar
| Category | Male-Crc | Female-Crc | Male-Rc | Female-Rc |
|---|---|---|---|---|
| 2.9166666666666665 | 0.0 | 0.0 | 0.0 | 0.0 |
| 3.5 | 0.0 | 0.0 | 0.0 | 0.0 |
| 4.5 | 0.0 | 0.0 | 0.0 | 0.0 |
| 5.5 | 8.108108108108109 | 5.714285714285714 | 0.0 | 0.0 |
| 6.5 | 39.189189189189186 | 34.285714285714285 | 0.0 | 0.0 |
| 7.5 | 72.97297297297297 | 82.85714285714286 | 0.0 | 0.0 |
| 8.5 | 100.0 | 100.0 | 0.0 | 2.1739130434782608 |
| 9.5 | 100.0 | 100.0 | 6.185567010309279 | 4.3478260869565215 |
| 10.5 | 100.0 | 100.0 | 18.556701030927837 | 30.434782608695656 |
| 11.5 | 100.0 | 100.0 | 50.51546391752577 | 72.82608695652173 |
| 12.5 | 100.0 | 100.0 | 92.78350515463917 | 89.13043478260869 |
| 13.5 | 100.0 | 100.0 | 100.0 | 97.82608695652173 |
| 14.5 | 100.0 | 100.0 | 100.0 | 100.0 |
| 15.5 | 100.0 | 100.0 | 100.0 | 100.0 |
| 16.5 | 100.0 | 100.0 | 100.0 | 100.0 |
| 17.5 | 100.0 | 100.0 | 100.0 | 100.0 |
| 18.5 | 100.0 | 100.0 | 100.0 | 100.0 |
| 18.916666666666668 | 100.0 | 100.0 | 100.0 | 100.0 |
### Chart: Lower first premolar
| Category | Male-Crc | Female-Crc | Male-Rc | Female-Rc |
|---|---|---|---|---|
| 2.9166666666666665 | 0.0 | 0.0 | 0.0 | 0.0 |
| 3.5 | 0.0 | 0.0 | 0.0 | 0.0 |
| 4.5 | 0.0 | 0.0 | 0.0 | 0.0 |
| 5.5 | 3.75 | 14.285714285714285 | 0.0 | 0.0 |
| 6.5 | 31.25 | 55.952380952380956 | 0.0 | 0.0 |
| 7.5 | 71.25 | 92.85714285714286 | 0.0 | 0.0 |
| 8.5 | 100.0 | 100.0 | 0.0 | 0.0 |
| 9.5 | 100.0 | 100.0 | 0.0 | 0.0 |
| 10.5 | 100.0 | 100.0 | 8.823529411764707 | 16.455696202531644 |
| 11.5 | 100.0 | 100.0 | 45.09803921568628 | 53.16455696202531 |
| 12.5 | 100.0 | 100.0 | 77.45098039215686 | 89.87341772151899 |
| 13.5 | 100.0 | 100.0 | 95.09803921568627 | 98.73417721518987 |
| 14.5 | 100.0 | 100.0 | 100.0 | 100.0 |
| 15.5 | 100.0 | 100.0 | 100.0 | 100.0 |
| 16.5 | 100.0 | 100.0 | 100.0 | 100.0 |
| 17.5 | 100.0 | 100.0 | 100.0 | 100.0 |
| 18.5 | 100.0 | 100.0 | 100.0 | 100.0 |
| 18.916666666666668 | 100.0 | 100.0 | 100.0 | 100.0 |（%）
（%）
### Chart: Upper second premolar
| Category | Male-Crc | Female-Crc | Male-Rc | Female-Rc |
|---|---|---|---|---|
| 3 | 0.0 | 0.0 | 0.0 | 0.0 |
| 3.5 | 0.0 | 0.0 | 0.0 | 0.0 |
| 4.5 | 0.0 | 0.0 | 0.0 | 0.0 |
| 5.5 | 1.4285714285714286 | 0.0 | 0.0 | 0.0 |
| 6.5 | 5.714285714285714 | 5.714285714285714 | 0.0 | 0.0 |
| 7.5 | 25.71428571428571 | 60.0 | 0.0 | 0.0 |
| 8.5 | 68.57142857142857 | 91.42857142857143 | 0.0 | 0.0 |
| 9.5 | 95.71428571428572 | 100.0 | 0.0 | 0.0 |
| 10.5 | 100.0 | 100.0 | 2.1739130434782608 | 9.63855421686747 |
| 11.5 | 100.0 | 100.0 | 26.08695652173913 | 24.096385542168676 |
| 12.5 | 100.0 | 100.0 | 57.608695652173914 | 50.602409638554214 |
| 13.5 | 100.0 | 100.0 | 85.86956521739131 | 77.10843373493977 |
| 14.5 | 100.0 | 100.0 | 100.0 | 92.7710843373494 |
| 15.5 | 100.0 | 100.0 | 100.0 | 97.59036144578313 |
| 16.5 | 100.0 | 100.0 | 100.0 | 100.0 |
| 17.5 | 100.0 | 100.0 | 100.0 | 100.0 |
| 18.5 | 100.0 | 100.0 | 100.0 | 100.0 |
| 18.916666666666668 | 100.0 | 100.0 | 100.0 | 100.0 |
### Chart: Lower second premolar
| Category | Male-Crc | Female-Crc | Male-Rc | Female-Rc |
|---|---|---|---|---|
| 3 | 0.0 | 0.0 | 0.0 | 0.0 |
| 3.5 | 0.0 | 0.0 | 0.0 | 0.0 |
| 4.5 | 0.0 | 0.0 | 0.0 | 0.0 |
| 5.5 | 0.0 | 0.0 | 0.0 | 0.0 |
| 6.5 | 14.492753623188406 | 16.417910447761194 | 0.0 | 0.0 |
| 7.5 | 34.78260869565217 | 59.70149253731343 | 0.0 | 0.0 |
| 8.5 | 79.71014492753623 | 98.50746268656717 | 0.0 | 0.0 |
| 9.5 | 97.10144927536231 | 100.0 | 0.0 | 0.0 |
| 10.5 | 100.0 | 100.0 | 1.9801980198019802 | 13.26530612244898 |
| 11.5 | 100.0 | 100.0 | 22.772277227722775 | 26.53061224489796 |
| 12.5 | 100.0 | 100.0 | 50.495049504950494 | 54.08163265306123 |
| 13.5 | 100.0 | 100.0 | 75.24752475247524 | 76.53061224489795 |
| 14.5 | 100.0 | 100.0 | 98.01980198019803 | 97.95918367346938 |
| 15.5 | 100.0 | 100.0 | 100.0 | 100.0 |
| 16.5 | 100.0 | 100.0 | 100.0 | 100.0 |
| 17.5 | 100.0 | 100.0 | 100.0 | 100.0 |
| 18.5 | 100.0 | 100.0 | 100.0 | 100.0 |
| 18.916666666666668 | 100.0 | 100.0 | 100.0 | 100.0 |（%）
（%）
Supplemental Figure 2
Cumulative frequency distribution of Crc and Rc in premolars. The Rc of the male upper first premolar showed negative skewness, but the other curves were very close to normal. Crc: crown complete; Rc: root length complete.
