# Supplementary material for: Estimation of dental age based on the developmental stages of permanent teeth in Japanese children and adolescents
Source: Sci Rep. 2022 Feb 28;12:3345. doi: 10.1038/s41598-022-07304-2 (PMC8885679; doi:10.1038/s41598-022-07304-2)
Supplement: Supplementary file 2 — Supplementary Information 2. [file 41598_2022_7304_MOESM2_ESM.pptx]

## Slide 1
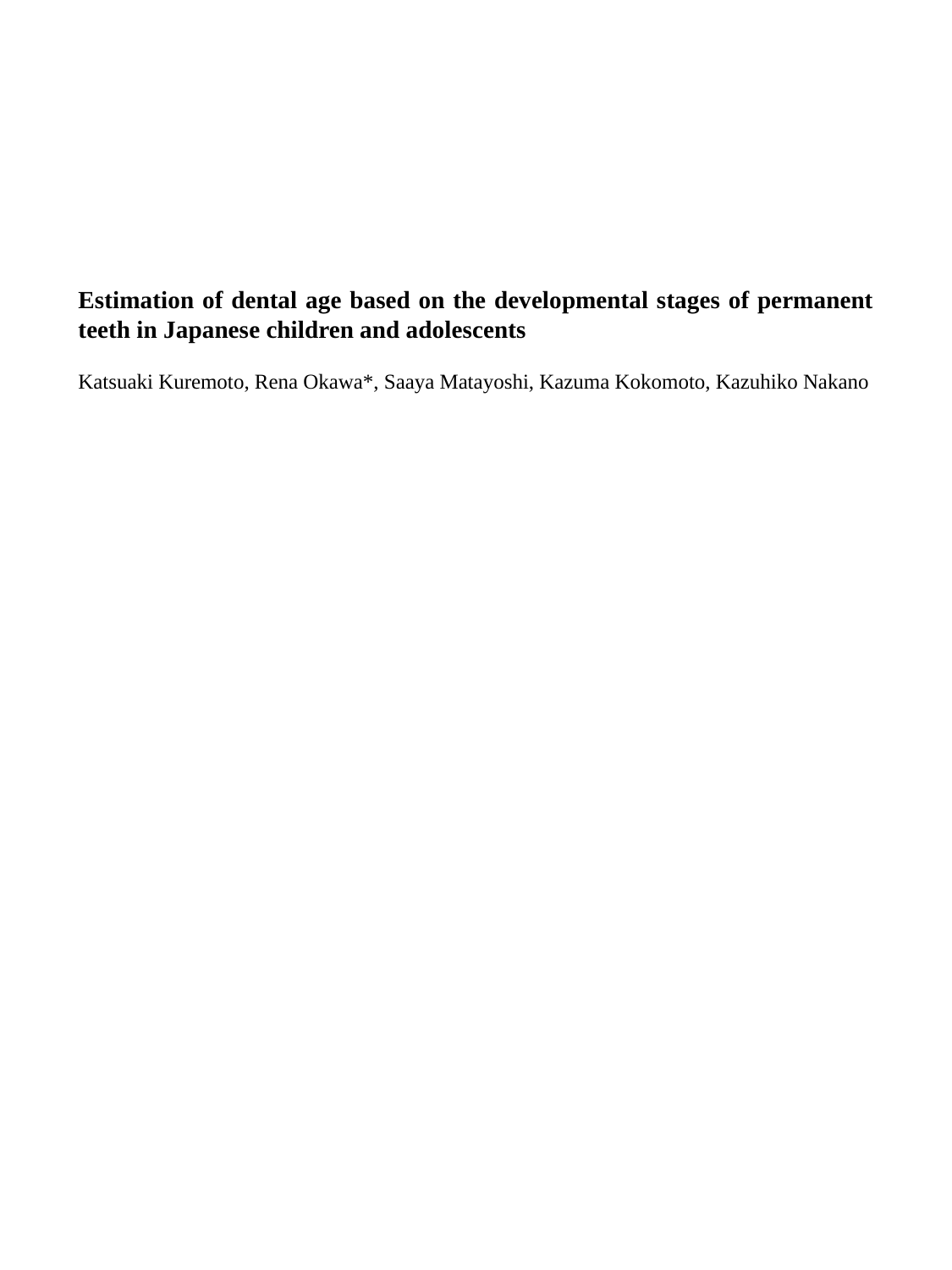

Estimation of dental age based on the developmental stages of permanent teeth in Japanese children and adolescents
Katsuaki Kuremoto, Rena Okawa*, Saaya Matayoshi, Kazuma Kokomoto, Kazuhiko Nakano

## Slide 2
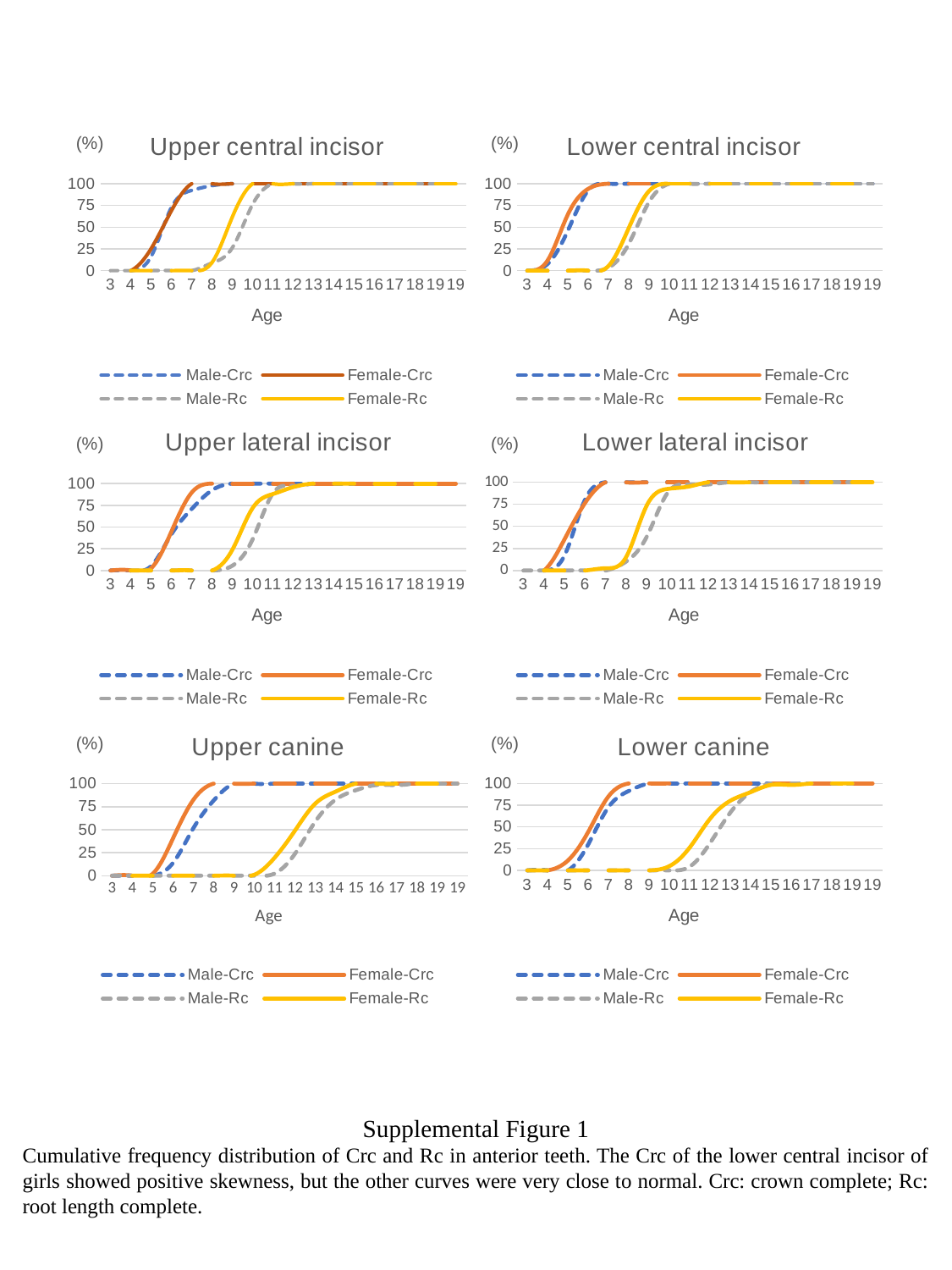

### Chart: Upper central incisor
| Category | Male-Crc | Female-Crc | Male-Rc | Female-Rc |
|---|---|---|---|---|
| 2.9166666666666665 | 0.0 | 0.0 | 0.0 | 0.0 |
| 3.5 | 0.0 | 0.0 | 0.0 | 0.0 |
| 4.5 | 16.304347826086957 | 25.352112676056336 | 0.0 | 0.0 |
| 5.5 | 72.82608695652173 | 69.01408450704226 | 0.0 | 0.0 |
| 6.5 | 92.3913043478261 | 100.0 | 0.0 | 0.0 |
| 7.5 | 97.82608695652173 | 100.0 | 8.823529411764707 | 9.523809523809524 |
| 8.5 | 100.0 | 100.0 | 26.47058823529412 | 61.904761904761905 |
| 9.5 | 100.0 | 100.0 | 76.47058823529412 | 100.0 |
| 10.5 | 100.0 | 100.0 | 100.0 | 100.0 |
| 11.5 | 100.0 | 100.0 | 100.0 | 100.0 |
| 12.5 | 100.0 | 100.0 | 100.0 | 100.0 |
| 13.5 | 100.0 | 100.0 | 100.0 | 100.0 |
| 14.5 | 100.0 | 100.0 | 100.0 | 100.0 |
| 15.5 | 100.0 | 100.0 | 100.0 | 100.0 |
| 16.5 | 100.0 | 100.0 | 100.0 | 100.0 |
| 17.5 | 100.0 | 100.0 | 100.0 | 100.0 |
| 18.5 | 100.0 | 100.0 | 100.0 | 100.0 |
| 18.916666666666668 | 100.0 | 100.0 | 100.0 | 100.0 |
### Chart: Lower central incisor
| Category | Male-Crc | Female-Crc | Male-Rc | Female-Rc |
|---|---|---|---|---|
| 2.9166666666666665 | 0.0 | 0.0 | 0.0 | 0.0 |
| 3.5 | 7.6923076923076925 | 11.76470588235294 | 0.0 | 0.0 |
| 4.5 | 46.15384615384615 | 64.70588235294117 | 0.0 | 0.0 |
| 5.5 | 92.3076923076923 | 94.11764705882352 | 0.0 | 0.0 |
| 6.5 | 100.0 | 100.0 | 3.4482758620689653 | 5.47945205479452 |
| 7.5 | 100.0 | 100.0 | 31.03448275862069 | 49.31506849315068 |
| 8.5 | 100.0 | 100.0 | 79.3103448275862 | 91.78082191780823 |
| 9.5 | 100.0 | 100.0 | 100.0 | 100.0 |
| 10.5 | 100.0 | 100.0 | 100.0 | 100.0 |
| 11.5 | 100.0 | 100.0 | 100.0 | 100.0 |
| 12.5 | 100.0 | 100.0 | 100.0 | 100.0 |
| 13.5 | 100.0 | 100.0 | 100.0 | 100.0 |
| 14.5 | 100.0 | 100.0 | 100.0 | 100.0 |
| 15.5 | 100.0 | 100.0 | 100.0 | 100.0 |
| 16.5 | 100.0 | 100.0 | 100.0 | 100.0 |
| 17.5 | 100.0 | 100.0 | 100.0 | 100.0 |
| 18.5 | 100.0 | 100.0 | 100.0 | 100.0 |
| 18.916666666666668 | 100.0 | 100.0 | 100.0 | 100.0 |(%)
(%)
### Chart: Upper lateral incisor
| Category | Male-Crc | Female-Crc | Male-Rc | Female-Rc |
|---|---|---|---|---|
| 2.9166666666666665 | 0.0 | 0.0 | 0.0 | 0.0 |
| 3.5 | 0.0 | 0.0 | 0.0 | 0.0 |
| 4.5 | 5.063291139240507 | 2.564102564102564 | 0.0 | 0.0 |
| 5.5 | 41.77215189873418 | 44.871794871794876 | 0.0 | 0.0 |
| 6.5 | 70.88607594936708 | 89.74358974358975 | 0.0 | 0.0 |
| 7.5 | 92.40506329113924 | 100.0 | 0.0 | 0.0 |
| 8.5 | 100.0 | 100.0 | 5.555555555555555 | 24.0 |
| 9.5 | 100.0 | 100.0 | 36.11111111111111 | 72.0 |
| 10.5 | 100.0 | 100.0 | 88.88888888888889 | 88.0 |
| 11.5 | 100.0 | 100.0 | 97.22222222222221 | 96.0 |
| 12.5 | 100.0 | 100.0 | 100.0 | 100.0 |
| 13.5 | 100.0 | 100.0 | 100.0 | 100.0 |
| 14.5 | 100.0 | 100.0 | 100.0 | 100.0 |
| 15.5 | 100.0 | 100.0 | 100.0 | 100.0 |
| 16.5 | 100.0 | 100.0 | 100.0 | 100.0 |
| 17.5 | 100.0 | 100.0 | 100.0 | 100.0 |
| 18.5 | 100.0 | 100.0 | 100.0 | 100.0 |
| 18.916666666666668 | 100.0 | 100.0 | 100.0 | 100.0 |
### Chart: Lower lateral incisor
| Category | Male-Crc | Female-Crc | Male-Rc | Female-Rc |
|---|---|---|---|---|
| 2.9166666666666665 | 0.0 | 0.0 | 0.0 | 0.0 |
| 3.5 | 0.0 | 0.0 | 0.0 | 0.0 |
| 4.5 | 16.901408450704224 | 35.294117647058826 | 0.0 | 0.0 |
| 5.5 | 80.28169014084507 | 76.47058823529412 | 0.0 | 0.0 |
| 6.5 | 100.0 | 100.0 | 0.0 | 2.5316455696202533 |
| 7.5 | 100.0 | 100.0 | 10.126582278481013 | 15.18987341772152 |
| 8.5 | 100.0 | 100.0 | 37.9746835443038 | 73.41772151898735 |
| 9.5 | 100.0 | 100.0 | 87.34177215189874 | 92.40506329113924 |
| 10.5 | 100.0 | 100.0 | 97.46835443037975 | 94.9367088607595 |
| 11.5 | 100.0 | 100.0 | 97.46835443037975 | 100.0 |
| 12.5 | 100.0 | 100.0 | 100.0 | 100.0 |
| 13.5 | 100.0 | 100.0 | 100.0 | 100.0 |
| 14.5 | 100.0 | 100.0 | 100.0 | 100.0 |
| 15.5 | 100.0 | 100.0 | 100.0 | 100.0 |
| 16.5 | 100.0 | 100.0 | 100.0 | 100.0 |
| 17.5 | 100.0 | 100.0 | 100.0 | 100.0 |
| 18.5 | 100.0 | 100.0 | 100.0 | 100.0 |
| 18.916666666666668 | 100.0 | 100.0 | 100.0 | 100.0 |(%)
(%)
### Chart: Lower canine
| Category | Male-Crc | Female-Crc | Male-Rc | Female-Rc |
|---|---|---|---|---|
| 2.9166666666666665 | 0.0 | 0.0 | 0.0 | 0.0 |
| 3.5 | 0.0 | 0.0 | 0.0 | 0.0 |
| 4.5 | 0.0 | 11.363636363636363 | 0.0 | 0.0 |
| 5.5 | 30.0 | 44.31818181818182 | 0.0 | 0.0 |
| 6.5 | 72.85714285714285 | 85.22727272727273 | 0.0 | 0.0 |
| 7.5 | 91.42857142857143 | 100.0 | 0.0 | 0.0 |
| 8.5 | 100.0 | 100.0 | 0.0 | 0.0 |
| 9.5 | 100.0 | 100.0 | 0.0 | 4.958677685950414 |
| 10.5 | 100.0 | 100.0 | 4.385964912280701 | 26.446280991735538 |
| 11.5 | 100.0 | 100.0 | 31.57894736842105 | 59.50413223140496 |
| 12.5 | 100.0 | 100.0 | 66.66666666666666 | 80.16528925619835 |
| 13.5 | 100.0 | 100.0 | 90.35087719298247 | 90.08264462809917 |
| 14.5 | 100.0 | 100.0 | 100.0 | 98.34710743801654 |
| 15.5 | 100.0 | 100.0 | 100.0 | 98.34710743801654 |
| 16.5 | 100.0 | 100.0 | 100.0 | 100.0 |
| 17.5 | 100.0 | 100.0 | 100.0 | 100.0 |
| 18.5 | 100.0 | 100.0 | 100.0 | 100.0 |
| 18.916666666666668 | 100.0 | 100.0 | 100.0 | 100.0 |
### Chart: Upper canine
| Category | Male-Crc | Female-Crc | Male-Rc | Female-Rc |
|---|---|---|---|---|
| 2.9166666666666665 | 0.0 | 0.0 | 0.0 | 0.0 |
| 3.5 | 0.0 | 0.0 | 0.0 | 0.0 |
| 4.5 | 0.0 | 2.197802197802198 | 0.0 | 0.0 |
| 5.5 | 13.978494623655912 | 40.65934065934066 | 0.0 | 0.0 |
| 6.5 | 51.61290322580645 | 82.41758241758241 | 0.0 | 0.0 |
| 7.5 | 81.72043010752688 | 100.0 | 0.0 | 0.0 |
| 8.5 | 100.0 | 100.0 | 0.0 | 0.0 |
| 9.5 | 100.0 | 100.0 | 0.0 | 1.0869565217391304 |
| 10.5 | 100.0 | 100.0 | 2.380952380952381 | 19.565217391304348 |
| 11.5 | 100.0 | 100.0 | 23.809523809523807 | 48.91304347826087 |
| 12.5 | 100.0 | 100.0 | 58.730158730158735 | 78.26086956521739 |
| 13.5 | 100.0 | 100.0 | 82.53968253968253 | 91.30434782608695 |
| 14.5 | 100.0 | 100.0 | 92.85714285714286 | 100.0 |
| 15.5 | 100.0 | 100.0 | 98.4126984126984 | 100.0 |
| 16.5 | 100.0 | 100.0 | 98.4126984126984 | 100.0 |
| 17.5 | 100.0 | 100.0 | 100.0 | 100.0 |
| 18.5 | 100.0 | 100.0 | 100.0 | 100.0 |
| 18.916666666666668 | 100.0 | 100.0 | 100.0 | 100.0 |(%)
(%)
Supplemental Figure 1
Cumulative frequency distribution of Crc and Rc in anterior teeth. The Crc of the lower central incisor of girls showed positive skewness, but the other curves were very close to normal. Crc: crown complete; Rc: root length complete.
