# Supplementary material for: Estimation of dental age based on the developmental stages of permanent teeth in Japanese children and adolescents
Source: Sci Rep. 2022 Feb 28;12:3345. doi: 10.1038/s41598-022-07304-2 (PMC8885679; doi:10.1038/s41598-022-07304-2)
Supplement: Supplementary file 3 — Supplementary Information 3. [file 41598_2022_7304_MOESM3_ESM.pptx]

## Slide 1
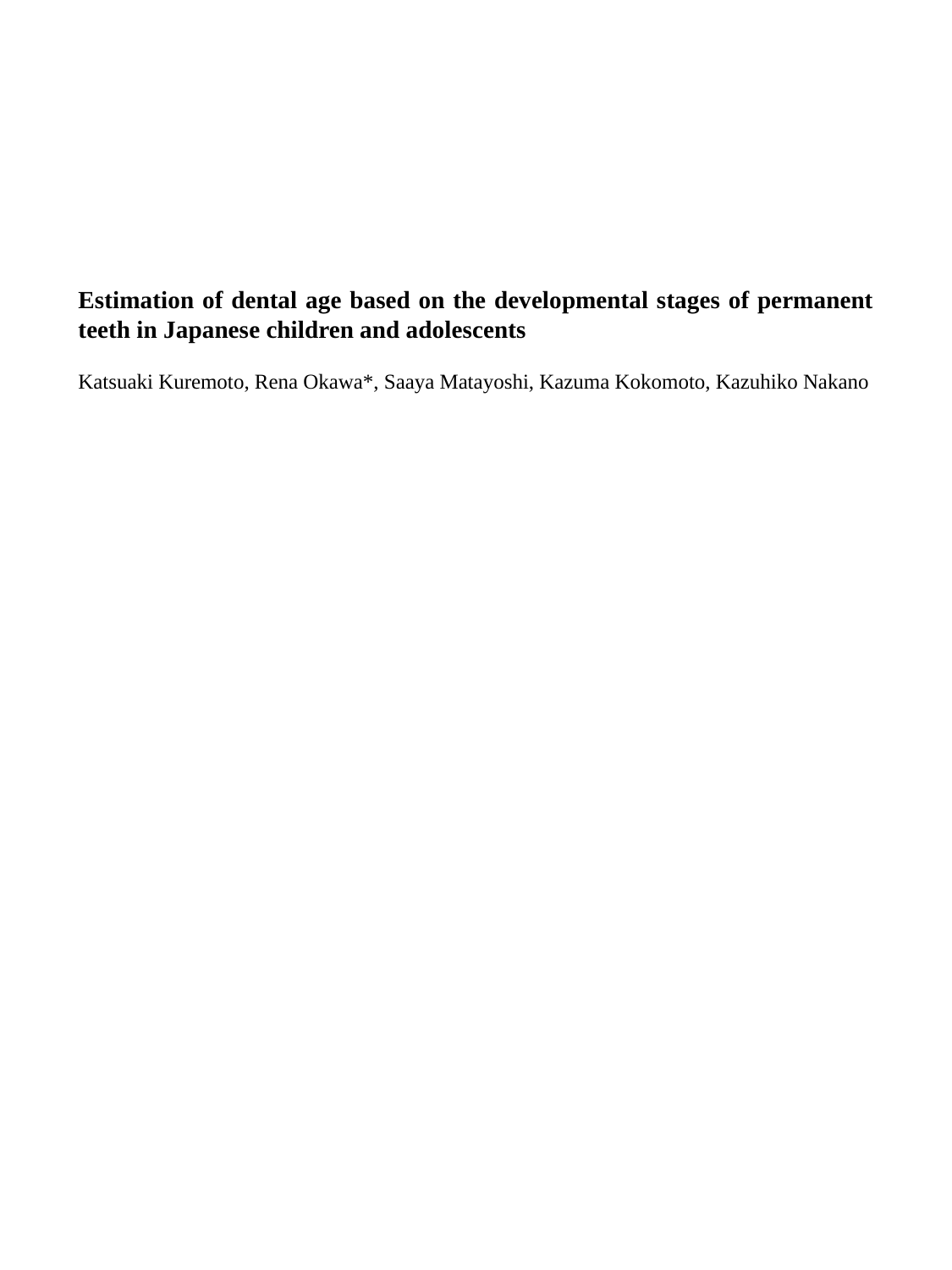

Estimation of dental age based on the developmental stages of permanent teeth in Japanese children and adolescents
Katsuaki Kuremoto, Rena Okawa*, Saaya Matayoshi, Kazuma Kokomoto, Kazuhiko Nakano

## Slide 2
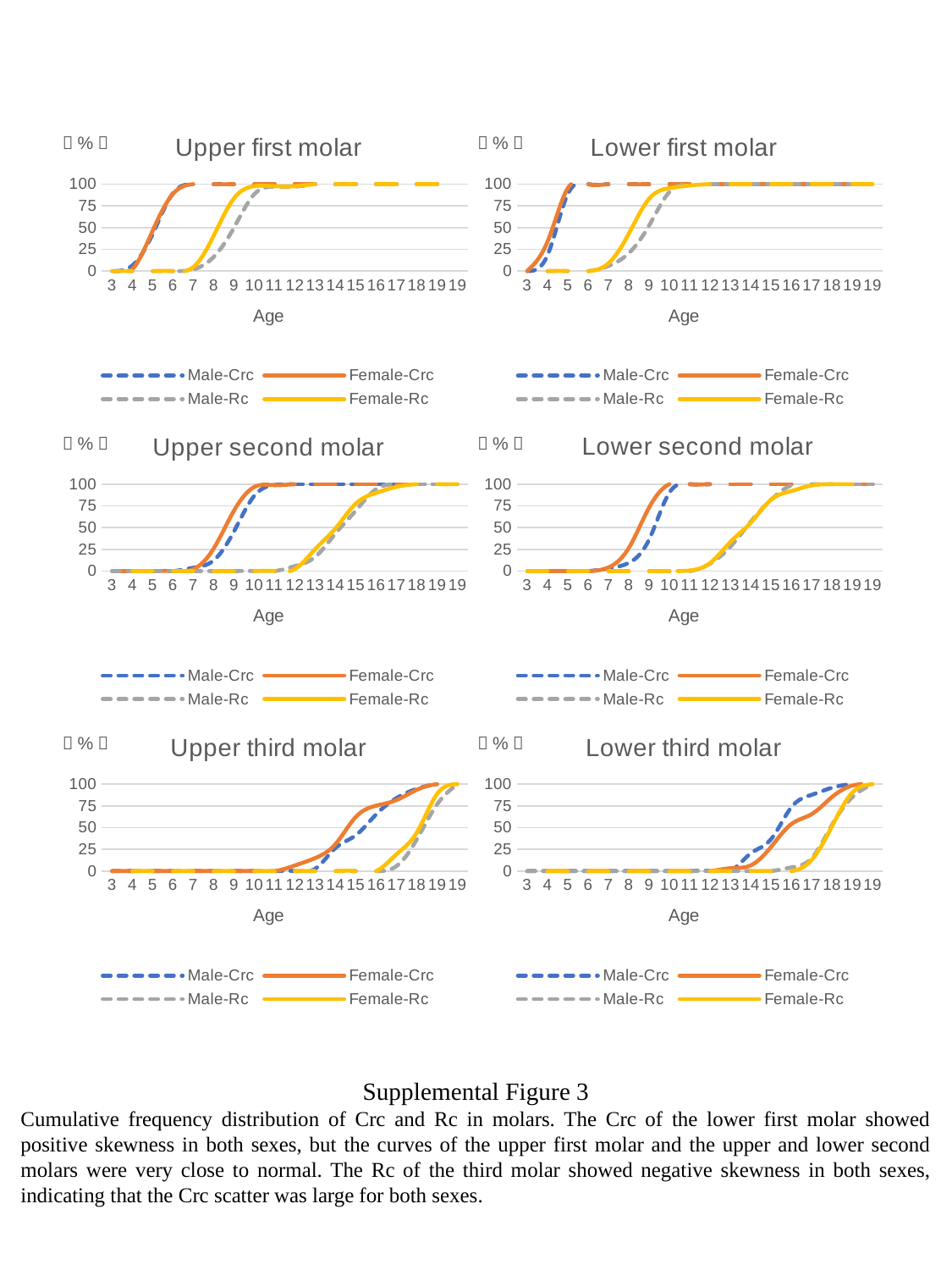

### Chart: Upper first molar
| Category | Male-Crc | Female-Crc | Male-Rc | Female-Rc |
|---|---|---|---|---|
| 2.9166666666666665 | 0.0 | 0.0 | 0.0 | 0.0 |
| 3.5 | 6.493506493506493 | 2.2222222222222223 | 0.0 | 0.0 |
| 4.5 | 42.857142857142854 | 46.666666666666664 | 0.0 | 0.0 |
| 5.5 | 89.6103896103896 | 88.88888888888889 | 0.0 | 0.0 |
| 6.5 | 100.0 | 100.0 | 1.9230769230769231 | 4.651162790697675 |
| 7.5 | 100.0 | 100.0 | 16.346153846153847 | 40.69767441860465 |
| 8.5 | 100.0 | 100.0 | 50.0 | 83.72093023255815 |
| 9.5 | 100.0 | 100.0 | 88.46153846153845 | 97.67441860465115 |
| 10.5 | 100.0 | 100.0 | 97.11538461538461 | 97.67441860465115 |
| 11.5 | 100.0 | 100.0 | 97.11538461538461 | 97.67441860465115 |
| 12.5 | 100.0 | 100.0 | 100.0 | 100.0 |
| 13.5 | 100.0 | 100.0 | 100.0 | 100.0 |
| 14.5 | 100.0 | 100.0 | 100.0 | 100.0 |
| 15.5 | 100.0 | 100.0 | 100.0 | 100.0 |
| 16.5 | 100.0 | 100.0 | 100.0 | 100.0 |
| 17.5 | 100.0 | 100.0 | 100.0 | 100.0 |
| 18.5 | 100.0 | 100.0 | 100.0 | 100.0 |
| 18.916666666666668 | 100.0 | 100.0 | 100.0 | 100.0 |
### Chart: Lower first molar
| Category | Male-Crc | Female-Crc | Male-Rc | Female-Rc |
|---|---|---|---|---|
| 3 | 0.0 | 0.0 | 0.0 | 0.0 |
| 3.5 | 18.51851851851852 | 34.090909090909086 | 0.0 | 0.0 |
| 4.5 | 88.88888888888889 | 95.45454545454545 | 0.0 | 0.0 |
| 5.5 | 100.0 | 100.0 | 0.0 | 0.0 |
| 6.5 | 100.0 | 100.0 | 5.9259259259259265 | 9.090909090909092 |
| 7.5 | 100.0 | 100.0 | 20.74074074074074 | 43.18181818181818 |
| 8.5 | 100.0 | 100.0 | 52.59259259259259 | 83.33333333333334 |
| 9.5 | 100.0 | 100.0 | 91.11111111111111 | 95.45454545454545 |
| 10.5 | 100.0 | 100.0 | 100.0 | 98.48484848484848 |
| 11.5 | 100.0 | 100.0 | 100.0 | 100.0 |
| 12.5 | 100.0 | 100.0 | 100.0 | 100.0 |
| 13.5 | 100.0 | 100.0 | 100.0 | 100.0 |
| 14.5 | 100.0 | 100.0 | 100.0 | 100.0 |
| 15.5 | 100.0 | 100.0 | 100.0 | 100.0 |
| 16.5 | 100.0 | 100.0 | 100.0 | 100.0 |
| 17.5 | 100.0 | 100.0 | 100.0 | 100.0 |
| 18.5 | 100.0 | 100.0 | 100.0 | 100.0 |
| 18.916666666666668 | 100.0 | 100.0 | 100.0 | 100.0 |（%）
（%）
### Chart: Upper second molar
| Category | Male-Crc | Female-Crc | Male-Rc | Female-Rc |
|---|---|---|---|---|
| 3 | 0.0 | 0.0 | 0.0 | 0.0 |
| 3.5 | 0.0 | 0.0 | 0.0 | 0.0 |
| 4.5 | 0.0 | 0.0 | 0.0 | 0.0 |
| 5.5 | 0.0 | 0.0 | 0.0 | 0.0 |
| 6.5 | 3.8095238095238098 | 2.1739130434782608 | 0.0 | 0.0 |
| 7.5 | 12.380952380952381 | 26.08695652173913 | 0.0 | 0.0 |
| 8.5 | 45.714285714285715 | 69.56521739130434 | 0.0 | 0.0 |
| 9.5 | 86.66666666666667 | 96.73913043478261 | 0.0 | 0.0 |
| 10.5 | 99.04761904761905 | 98.91304347826086 | 0.0 | 0.0 |
| 11.5 | 100.0 | 100.0 | 5.737704918032787 | 2.307692307692308 |
| 12.5 | 100.0 | 100.0 | 16.39344262295082 | 25.384615384615383 |
| 13.5 | 100.0 | 100.0 | 43.44262295081967 | 49.23076923076923 |
| 14.5 | 100.0 | 100.0 | 69.67213114754098 | 77.6923076923077 |
| 15.5 | 100.0 | 100.0 | 94.26229508196722 | 90.0 |
| 16.5 | 100.0 | 100.0 | 100.0 | 96.92307692307692 |
| 17.5 | 100.0 | 100.0 | 100.0 | 100.0 |
| 18.5 | 100.0 | 100.0 | 100.0 | 100.0 |
| 18.916666666666668 | 100.0 | 100.0 | 100.0 | 100.0 |
### Chart: Lower second molar
| Category | Male-Crc | Female-Crc | Male-Rc | Female-Rc |
|---|---|---|---|---|
| 2.9166666666666665 | 0.0 | 0.0 | 0.0 | 0.0 |
| 3.5 | 0.0 | 0.0 | 0.0 | 0.0 |
| 4.5 | 0.0 | 0.0 | 0.0 | 0.0 |
| 5.5 | 0.0 | 0.0 | 0.0 | 0.0 |
| 6.5 | 3.1746031746031744 | 4.225352112676056 | 0.0 | 0.0 |
| 7.5 | 9.523809523809524 | 26.76056338028169 | 0.0 | 0.0 |
| 8.5 | 36.507936507936506 | 73.23943661971832 | 0.0 | 0.0 |
| 9.5 | 90.47619047619048 | 100.0 | 0.0 | 0.0 |
| 10.5 | 100.0 | 100.0 | 0.0 | 0.7142857142857143 |
| 11.5 | 100.0 | 100.0 | 8.633093525179856 | 9.285714285714286 |
| 12.5 | 100.0 | 100.0 | 28.05755395683453 | 33.57142857142857 |
| 13.5 | 100.0 | 100.0 | 56.83453237410072 | 55.714285714285715 |
| 14.5 | 100.0 | 100.0 | 82.01438848920863 | 82.14285714285714 |
| 15.5 | 100.0 | 100.0 | 98.56115107913669 | 92.14285714285714 |
| 16.5 | 100.0 | 100.0 | 100.0 | 98.57142857142858 |
| 17.5 | 100.0 | 100.0 | 100.0 | 100.0 |
| 18.5 | 100.0 | 100.0 | 100.0 | 100.0 |
| 18.916666666666668 | 100.0 | 100.0 | 100.0 | 100.0 |（%）
（%）
### Chart: Upper third molar
| Category | Male-Crc | Female-Crc | Male-Rc | Female-Rc |
|---|---|---|---|---|
| 2.9166666666666665 | 0.0 | 0.0 | 0.0 | 0.0 |
| 3.5 | 0.0 | 0.0 | 0.0 | 0.0 |
| 4.5 | 0.0 | 0.0 | 0.0 | 0.0 |
| 5.5 | 0.0 | 0.0 | 0.0 | 0.0 |
| 6.5 | 0.0 | 0.0 | 0.0 | 0.0 |
| 7.5 | 0.0 | 0.0 | 0.0 | 0.0 |
| 8.5 | 0.0 | 0.0 | 0.0 | 0.0 |
| 9.5 | 0.0 | 0.0 | 0.0 | 0.0 |
| 10.5 | 0.0 | 0.0 | 0.0 | 0.0 |
| 11.5 | 0.0 | 6.451612903225806 | 0.0 | 0.0 |
| 12.5 | 2.666666666666667 | 15.053763440860216 | 0.0 | 0.0 |
| 13.5 | 26.666666666666668 | 31.182795698924732 | 0.0 | 0.0 |
| 14.5 | 41.333333333333336 | 62.365591397849464 | 0.0 | 0.0 |
| 15.5 | 65.33333333333333 | 75.26881720430107 | 0.0 | 0.0 |
| 16.5 | 84.0 | 81.72043010752688 | 5.769230769230769 | 19.444444444444446 |
| 17.5 | 94.66666666666667 | 93.54838709677419 | 36.53846153846153 | 44.44444444444444 |
| 18.5 | 100.0 | 100.0 | 76.92307692307693 | 88.88888888888889 |
| 18.916666666666668 | 100.0 | 100.0 | 100.0 | 100.0 |
### Chart: Lower third molar
| Category | Male-Crc | Female-Crc | Male-Rc | Female-Rc |
|---|---|---|---|---|
| 2.9166666666666665 | 0.0 | 0.0 | 0.0 | 0.0 |
| 3.5 | 0.0 | 0.0 | 0.0 | 0.0 |
| 4.5 | 0.0 | 0.0 | 0.0 | 0.0 |
| 5.5 | 0.0 | 0.0 | 0.0 | 0.0 |
| 6.5 | 0.0 | 0.0 | 0.0 | 0.0 |
| 7.5 | 0.0 | 0.0 | 0.0 | 0.0 |
| 8.5 | 0.0 | 0.0 | 0.0 | 0.0 |
| 9.5 | 0.0 | 0.0 | 0.0 | 0.0 |
| 10.5 | 0.0 | 0.0 | 0.0 | 0.0 |
| 11.5 | 0.0 | 0.0 | 0.0 | 0.0 |
| 12.5 | 0.0 | 3.278688524590164 | 0.0 | 0.0 |
| 13.5 | 20.408163265306122 | 6.557377049180328 | 0.0 | 0.0 |
| 14.5 | 36.734693877551024 | 27.86885245901639 | 0.0 | 0.0 |
| 15.583333333333334 | 73.46938775510205 | 54.09836065573771 | 4.25531914893617 | 0.0 |
| 16.5 | 87.75510204081633 | 65.57377049180327 | 14.893617021276595 | 12.903225806451612 |
| 17.5 | 95.91836734693877 | 85.24590163934425 | 53.191489361702125 | 51.61290322580645 |
| 18.5 | 100.0 | 98.36065573770492 | 85.1063829787234 | 90.32258064516128 |
| 18.916666666666668 | 100.0 | 100.0 | 100.0 | 100.0 |（%）
（%）
Supplemental Figure 3
Cumulative frequency distribution of Crc and Rc in molars. The Crc of the lower first molar showed positive skewness in both sexes, but the curves of the upper first molar and the upper and lower second molars were very close to normal. The Rc of the third molar showed negative skewness in both sexes, indicating that the Crc scatter was large for both sexes.
