# Supplementary material for: Estimation of dental age based on the developmental stages of permanent teeth in Japanese children and adolescents
Source: Sci Rep. 2022 Feb 28;12:3345. doi: 10.1038/s41598-022-07304-2 (PMC8885679; doi:10.1038/s41598-022-07304-2)
Supplement: Supplementary file 4 — Supplementary Information 4. [file 41598_2022_7304_MOESM4_ESM.docx]

**Estimation of dental age based on the developmental stages of permanent teeth in Japanese children and adolescents**

Katsuaki Kuremoto, Rena Okawa*, Saaya Matayoshi, Kazuma Kokomoto, Kazuhiko Nakano

**Supplemental Table 1 Reliability and correlation for the total score**

|  | Total variance of tooth developmental score by samples | Variance of total tooth developmental score by permanent teeth | Cronbach’s Alpha |
| --- | --- | --- | --- |
|  |  |  |  |
| Male | 123.328 | 1645.338 | 0.987 |
| Female | 117.422 | 1546.920 | 0.986 |
|  |  |  |  |
